# Central Venous Pressure and Clinical Outcomes During Left-Sided Mechanical Support for Acute Myocardial Infarction and Cardiogenic Shock

**DOI:** 10.3389/fcvm.2020.00155

**Published:** 2020-08-28

**Authors:** Evan H. Whitehead, Katherine L. Thayer, Daniel Burkhoff, Nir Uriel, E. Magnus Ohman, William O'Neill, Navin K. Kapur

**Affiliations:** ^1^Tufts Medical Center, Cardiovascular Center for Research and Innovation, Boston, MA, United States; ^2^Cardiovascular Research Foundation, New York, NY, United States; ^3^New York-Presbyterian, Advanced Heart Failure and Cardiac Transplant, New York, NY, United States; ^4^Duke Medical Center, Durham, NC, United States; ^5^Henry Ford Health System, Detroit, MI, United States

**Keywords:** central venous pressure, right heart failure, Impella RP, cardiogenic shock, mechanical circulatory support

## Abstract

**Background:** Right ventricular failure (RVF) is associated with increased mortality among patients receiving left ventricular mechanical circulatory support (LV-MCS) for cardiogenic shock and requires prompt recognition and management. Increased central venous pressure (CVP) is an indicator of potential RVF.

**Objectives:** We studied whether elevated CVP during LV-MCS for acute myocardial infarction complicated by cardiogenic shock is associated with higher mortality.

**Methods:** Between January 2014 and June 2019, we analyzed hemodynamic parameters during Impella LV-MCS from 28 centers in the United States participating in the global, prospective catheter-based ventricular assist device (cVAD) study. A total of 132 patients with a documented CVP measurement while on Impella left-sided support for cardiogenic shock were identified.

**Results:** CVP was significantly higher among patients who died in the hospital (14.0 vs. 11.7 mmHg, *p* = 0.014), and a CVP >12 identified patients at significantly higher risk for in-hospital mortality (65 vs. 45%, *p* = 0.02). CVP remained significantly associated with in-hospital mortality even after adjustment in a multivariable model (adjusted OR 1.10 [95% CI 1.02–1.19] per 1 mmHg increase). LV-MCS suction events were non-significantly more frequent among patients with high vs. low CVP (62.11 vs. 7.14 events, *p* = 0.067).

**Conclusion:** CVP is a single, readily accessible hemodynamic parameter which predicts a higher rate of short-term mortality and may identify subclinical RVF in patients receiving LV-MCS for cardiogenic shock.

## Introduction

Right ventricular failure (RVF) after myocardial infarction, cardiotomy, or left-sided mechanical support is associated with increased morbidity and mortality (1). In these patients, early identification of RVF and deployment of temporary RV support may improve outcomes. Increased central venous pressure (CVP) measured before or during surgical left ventricular assist device (LVAD) support is a well-established clinical indicator of risk for RVF (2). The role for CVP in the setting of short-term mechanical circulatory support is less well-characterized.

The Recover Right (RR) Trial demonstrated the safety and potential efficacy of the Impella RP, a rapidly deployed percutaneous RV assist device, in the setting of cardiogenic shock. RVF in the RR Trial was defined as a cardiac index <2.2 L/min/m^2^ despite the continuous infusion of high dose inotropes and any of the following: a CVP >15 mmHg, CVP-to-pulmonary capillary wedge pressure (PCWP) ratio >0.63, or moderate to severe global RV dysfunction (3). The Impella RP post-approval study demonstrated improved survival among patients receiving the Impella RP who met the pre-market IDE RR inclusion criteria for RVF compared to those who did not meet these criteria and received the device as a salvage procedure. Furthermore, a recent analysis of the SHOCK Trial and Registry identified that 45 and 38% of patients would have met hemodynamic inclusion criteria for RVF in the RR Trial. These findings and other recent reports suggest that elevated CVP is an important indicator of RVF and early identification and management of RVF may improve outcomes (4).

No studies have explored a role for CVP monitoring in the setting of short-term left ventricular mechanical circulatory support (LV-MCS) with the Impella pump for cardiogenic shock (CS). Furthermore, deciding when to embark on an extensive, multimodality assessment for RV dysfunction remains clinically challenging. We hypothesized that CVP may be a sensitive, readily accessible indicator that could be used to trigger a comprehensive evaluation for RVF in patients receiving LV-MCS. In this study, we utilize data from the catheter-based ventricular assist device (cVAD) registry to assess the relationship between CVP, mortality, and indicators of RV failure among patients receiving left-sided Impella support.

## Methods

Between January 2014 and June 2019, we analyzed hemodynamic parameters during Impella LV-MCS from 28 centers in the United States participating in the global, prospective catheter-based ventricular assist device (cVAD) study (5). A total of 132 patients with a documented CVP measurement while on left-sided Impella support for acute myocardial infarction complicated by cardiogenic shock were identified. The diagnosis of acute myocardial infarction (AMI) was made by analysis of ECG changes, cardiac enzymes, and/or identification of an infarct-related coronary occlusion on emergency angiography. The central clinical events committee confirmed the presence of AMICS based on chart information collected. Cardiogenic shock was defined as a (1) systolic blood pressure ≤90 mm Hg or need for inotropes or vasopressors to maintain systolic blood pressures ≥90 mm Hg, (2) signs of peripheral hypoperfusion, and (3) cardiac index <2.2 L/min/m^2^ and pulmonary capillary wedge pressure ≥15 mm Hg. In order to evaluate the potential utility of CVP as a predictor of death and RV failure, we restricted our analysis to a subset of patients receiving LV-MCS for CS who had a documented CVP during support. When multiple CVP values were recorded prior to initiation of support, we used the value obtained closest to support initiation as the baseline CVP. When multiple CVP values were recorded during support, we report the average of those values as the CVP during support. Baseline characteristics including demographics and medical history as well as laboratory values, hemodynamic parameters and admission characteristics were obtained from the cVAD study. The primary endpoint of the study was in-hospital mortality, which was adjudicated in the registry by an independent clinical events committee.

As an additional validation cohort, a second analysis was performed among patients in the Impella Quality Assurance (IQ) database, a large, HIPAA compliant database of Impella patients maintained by the device manufacturer Abiomed, Inc. (6). Whereas, the cVAD registry contains a relatively small subset of patients with detailed patient information and independently adjudicated events, the IQ database captures nearly all patients treated with an Impella device in the United States but contains less in-depth patient information. Only death or survival to explant are available from the IQ database, so death prior to explant was used as the primary endpoint for the IQ database analysis rather than in-hospital mortality. Patients with AMICS with a CVP available during left-sided Impella support who were treated between October 2011 to June 2019 were identified from the IQ database using the same inclusion criteria as described above.

### Statistical Analyses

Continuous variables were reported as means and standard deviations and compared using independent *t*-tests, while categorical variables were reported as frequency and percentages and compared using Pearson chi-squared tests. Statistical significance was reported using an α level of 0.05. Laboratory values and hemodynamic parameters recorded during mechanical support were compared in the same fashion. To examine the association between mortality and CVP as a continuous variable, we constructed a univariate logistic regression model with in-hospital mortality as the dependent variable and CVP during Impella support as the independent variable. The resulting curve was plotted with 95% confidence limits per point. To determine the optimal cutoff value of CVP which best predicted mortality, we plotted the Receiver Operating Characteristic (ROC) curves of mortality and CVP and identified the optimal point as the point closest to a sensitivity and specificity of (0,0). The Youden index, Mathews correlation coefficient, and total accuracy were also maximized around the selected cutoff point. Various univariate logistic regression models were generated with in-hospital death as the outcome with baseline and procedural characteristics as independent predictors. Variables with statistically significant univariate odds ratios were then included in a multivariable logistic regression model to report adjusted odds ratio with 95% CI for in-hospital mortality.

## Results

### Baseline and Admission Characteristics

Out of 132 patients receiving LV-MCS for cardiogenic shock with available CVP data from the cVAD registry, 59 died in the hospital and 73 survived to discharge. Baseline characteristics, laboratory values, and hemodynamic parameters obtained before and after initiation of Impella support are displayed in Table 1. Hemodynamic data were more commonly measured after initiation of LV-MCS. Prior to initiation of LV-MCS, mean cardiac index (CI) was 1.9 ± 0.5 L/min/m^2^, pulmonary capillary wedge pressure (PCWP) was 26.5 ± 11.2 mmHg, and lactate was 6.0 ± 4.6 mmol/L. Compared to baseline values, CI improved significantly to 2.7 ± 0.9 L/min (*p* = 0.0001) and PCWP improved to 21.7 ± 8.7 mmHg (*p* = 0.09) with initiation of support. Admission and procedural characteristics are summarized in Table 2. Cardiogenic shock was due to STEMI in 72.2% and NSTEMI in 27.8% of patients, and the mean duration of Impella support was 92.7 ± 76.8 h. Significant differences between those who died in hospital and those who survived to discharge were noted in the rates of CPR (54.2 vs. 35.6%, *p* = 0.032) and mechanical ventilation (62.7 vs. 39.7%, *p* = 0.009).

**Table 1:** Baseline characteristics and laboratory values/hemodynamics before and during Impella support.

**Characteristics**	**All (*n* = 132) % (*n*)**	**Survived to discharge (*n* = 73) % (*n*)**	**Died in hospital (*n* = 59) % (*n*)**	***P*-value**
Age (mean)	63.1	61.4	65.3	0.044
Male	81.1 (107)	84.9 (62)	76.3 (45)	0.265
Race				
American Indian or Alaska Native	1.5 (2)	1.4 (1)	1.7 (1)	1.000
Asian	3.8 (5)	5.5 (4)	1.7 (1)	0.380
Black or African American	6.1 (8)	1.4 (1)	11.9 (7)	0.022
Caucasian	68.9 (91)	71.2 (52)	66.1 (39)	0.573
Native Hawaiian or Other Pacific Islander	0.0 (0)	0.0 (0)	0.0 (0)	–
Other	3.0 (4)	1.4 (1)	5.1 (3)	0.324
Unknown	16.7 (22)	19.2 (14)	13.6 (8)	0.484
BSA (m^2^) mean	2.0	2.0	2.0	0.361
**Medical history**	**% (*****n*****)**	**% (*****n*****)**	**% (*****n*****)**	***P*****-value**
Smoker	59.7 (74)	63.8 (44)	54.6 (30)	0.358
Hyperlipidemia	57.9 (73)	52.9 (37)	64.3 (36)	0.209
Hypertension	76.2 (99)	70.4 (50)	83.1(49)	0.103
Diabetes mellitus	48.8 (62)	45.8 (33)	52.7 (29)	0.477
CAD	50.0 (63)	52.9 (37)	46.4 (26)	0.591
Angina	28.7 (35)	29.8 (20)	27.3 (15)	0.842
Stroke/TIA	6.4 (8)	2.9 (2)	10.91 (6)	0.137
Renal Insufficiency	17.5 (22)	12.7 (9)	23.6 (13)	0.155
Dialysis	1.6 (2)	0.0 (0)	3.6 (2)	0.189
COPD/chronic pulmonary disease	18.6 (23)	21.7 (15)	14.6 (8)	0.358
Arrhythmia	18.6 (23)	15.7 (11)	22.2 (12)	0.364
PVD	10.4 (13)	7.0 (5)	14.8 (8)	0.236
CHF	19.1 (24)	12.7 (9)	27.3 (15)	0.043
NYHA class				
I	2.7 (1)	5.3 (1)	0.0 (0)	1.000
II	10.8 (4)	15.8 (3)	5.6 (1)	0.604
III	29.7 (11)	36.8 (7)	22.2 (4)	0.476
IV	56.8 (21)	42.1 (8)	72.2 (13)	0.099
III/IV	86.5 (32)	79.0 (15)	94.4 (17)	0.340
Valvular disease	9.6 (12)	7.1 (5)	12.7 (7)	0.365
Cardiomyopathy	12.8 (16)	9.9 (7)	16.7 (9)	0.289
Prior MI	37.0 (44)	36.8 (25)	37.3 (19)	1.000
Prior AICD/pacer implanted	6.1 (8)	4.2 (3)	8.5 (5)	0.466
Prior PCI	35.4 (45)	30.0 (21)	42.1 (24)	0.193
Prior CABG	9.2 (12)	8.3 (6)	10.3 (6)	0.766
LVEF (%)	25.1 (71)	21.6 (36)	28.7 (35)	0.021
**Laboratory values at baseline**	**Mean** **±** **SD**	**Mean** **±** **SD**	**Mean** **±** **SD**	***P*****-value**
Creatinine (mg/dL)	2.1 ± 6.0	2.3 ± 8.1	1.9 ± 1.4	0.676
BUN (mg/dL)	26.4 ± 17.9	25.0 ± 19.6	27.9 ± 16.0	0.394
Lactate (mmol/L)	6.0 ± 4.6	5.6 ± 4.2	6.4 ± 5.0	0.609
Total bilirubin (mg/dL)	1.6 ± 4.6	1.0 ± 1.1	2.2 ± 6.4	0.246
**Hemodynamics at baseline**				
HR (bpm)	96.1 ± 26.8 (119)	94.7 ± 26.4 (66)	97.7 ± 27.6 (53)	0.554
Systolic blood pressure (mmHg)	106.4 ± 23.8 (117)	105.9 ± 21.3 (66)	107.1 ± 26.8 (51)	0.791
Diastolic blood pressure (mmHg)	66.7 ± 18.3 (117)	67.5 ± 16.4 (66)	65.6 ± 20.6 (51)	0.588
Mean arterial pressure (mmHg)	80.6 ± 19.7 (117)	80.3 ± 17.8 (66)	80.9 ± 22.1 (51)	0.878
Cardiac index (L/min/m^2^)	1.9 ± 0.5 (22)	1.9 ± 0.5 (15)	2.0 ± 0.64(7)	0.572
Cardiac output (L/min)	3.7 ± 1.1 (22)	3.6 ± 1.1 (15)	4.1 ± 1.3 (7)	0.331
PCWP (mmHg)	26.5 ± 11.2 (14)	23.0 ± 11.3 (10)	35.3 ± 4.0 (4)	0.061
Pulmonary artery systolic pressure (mmHg)	38.3 ± 12.6 (27)	37.0 ± 13.2 (17)	40.6 ± 11.6 (10)	0.479
LVEDP (mmHg)	28.4 ± 10.3 (14)	27.5 ± 9.5 (8)	29.5 ± 12.0 (6)	0.733
CVP (mmHg)	13.5 ± 6.8 (23)	12.1 ± 5.4 (18)	18.5 ± 9.6 (5)	0.060
CVP/PCWP ratio	0.7 ± 0.5 (10)	0.7 ± 0.5 (9)	0.46 (1)	–
CVP/MAP ratio	0.2 ± 0.1 (21)	0.1 ± 0.1 (17)	0.3 ± 0.2 (4)	0.237
Mixed venous oxygen saturation (%)	53.33 ± 8.22 (9)	53.86 ± 9.37 (7)	51.50 ± 2.12 (2)	0.746
**Laboratory values during Impella support**				
Creatinine (mg/dL)	1.9 ± 1.2	1.6 ± 1.0	2.3 ± 1.4	0.005
BUN (mg/dL)	30.3 ± 16.8	27.3 ± 13.9	33.6 ± 19.2	0.051
Lactate (mmol/L)	4.3 ± 4.01	3.1 ± 3.1	5.8 ± 4.6	0.009
Total bilirubin (mg/dL)	1.82 ± 2.23	1.2 ± 0.8	2.54 ± 3.08	0.011
**Hemodynamics during Impella support**				
HR (bpm)	90.1 ± 18.3 (126)	87.6 ± 17.9 (70)	93.3 ± 18.4 (56)	0.080
Systolic blood pressure (mmHg)	104.8 ± 21.2 (122)	109.0 ± 19.56 (66)	99.8 ± 22.2 (56)	0.016
Diastolic blood pressure (mmHg)	68.5 ± 16.3 (122)	68.5 ± 15.8 (66)	68.5 ± 17.0 (56)	0.998
Mean arterial pressure (mmHg)	79.9 ± 16.7 (123)	81.3 ± 16.0 (67)	78.3 ± 17.4 (56)	0.325
Cardiac index (L/min/m^2^)	2.7 ± 0.9 (87)	2.8 ± 0.8 (56)	2.4 ± 0.9 (31)	0.070
Cardiac output (L/min)	5.3 ± 1.8 (87)	5.5 ± 1.8 (56)	4.9 ± 1.7 (31)	0.146
PCWP (mmHg)	21.7 ± 8.7 (44)	20.8 ± 8.9 (30)	23.6 ± 8.2 (14)	0.333
Pulmonary artery pressure systolic (mmHg)	34.4 ± 13.2 (118)	33.1 ± 10.5 (67)	36.0 ± 16.0 (51)	0.269
LVEDP (mmHg)	27.0 ± 12.2 (7)	20.7 ± 6.1 (3)	31.8 ± 14.2 (4)	0.268
CVP (mmHg)	12.7 ± 5.3 (132)	11.7 ± 4.6 (73)	14.0 ± 5.9 (59)	0.014
CVP/PCWP ratio	0.7 ± 0.3 (36)	0.7 ± 0.3 (25)	0.7 ± 0.3 (11)	0.745
CVP/MAP ratio	0.2 ± 0.1 (103)	0.2 ± 0.1 (51)	0.2 ± 0.1 (52)	0.008
Mixed venous oxygen saturation (%)	62.3 ± 10.4 (41)	63.4 ± 9.8 (28)	60.0 ± 11.7 (13)	0.339

**Table 2:** Admission and procedural characteristics.

**Characteristics**	**Subjects with CVP** **during Impella support** **(*N* = 132 patients)**	**Survived to discharge** **(*N* = 73 patients)**	**Died in hospital** **(*N* = 59 patients)**	***P*-value**
Patient was transferred from another hospital	37.88% (50/132)	39.73% (29/73)	35.59% (21/59)	0.626
Patient was supported with an IABP prior to Impella support	12.98% (17/131)	9.59% (7/73)	17.24% (10/58)	0.195
Shock was present on admission	52.27% (69/132)	52.05% (38/73)	52.54% (31/59)	0.956
Shock (Primary indication for Impella support)	100.00% (132/132)	100.00% (73/73)	100.00% (59/59)	–
**Duration of shock**				
<6 h	64.39% (85/132)	65.75% (48/73)	62.71% (37/59)	0.717
6–12 h	4.55% (6/132)	4.11% (3/73)	5.08% (3/59)	0.789
12–24 h	5.30% (7/132)	5.48% (4/73)	5.08% (3/59)	0.920
>24 h	9.85% (13/132)	5.48% (4/73)	15.25% (9/59)	0.061
**If shock, patient experienced any of the following**				
Anoxic brain damage	4.72% (5/106)	3.08% (2/65)	7.32% (3/41)	0.316
Cardiac arrest	49.24% (65/132)	42.47% (31/73)	57.63% (34/59)	0.083
**If shock, patient required any of the following**				
Mechanical ventilation	50.00% (66/132)	39.73% (29/73)	62.71% (37/59)	0.009
CPR	43.94% (58/132)	35.62% (26/73)	54.24% (32/59)	0.032
Acute myocardial infarction	100.00% (132/132)	100.00% (73/73)	100.00% (59/59)	–
STEMI	72.22% (91/126)	73.53% (50/68)	70.69% (41/58)	0.723
NSTEMI	27.78% (35/126)	26.47% (18/68)	29.31% (17/58)	0.723
ECMO	12.1% (16/132)	5.48% (4/73)	20.34% (12/59)	0.009
**Number of lesions treated**				
Mean ± SD (*N*)	2.03 ± 1.48 (103)	1.92 ± 1.48 (59)	2.18 ± 1.50 (44)	0.370
**Duration of index PCI procedure (hours)**				
Mean ± SD (*N*)	2.76 ± 5.61 (113)	2.39 ± 3.61 (64)	3.23 ± 7.47 (49)	0.475
**Duration of device support (hours)**				
Mean ± SD (*N*)	92.73 ± 76.77 (123)	92.97 ± 70.85 (67)	92.45 ± 83.96 (56)	0.970
**ICU stay (days)**				
Mean ± SD (*N*)	11.09 ± 12.78 (130)	12.53 ± 13.10 (72)	9.29 ± 12.24 (58)	0.152
**Duration of index hospitalization (days)**				
Mean ± SD (*N*)	13.25 ± 13.44 (126)	16.26 ± 13.07 (68)	9.72 ± 13.11 (58)	0.006

### Relationship Between CVP and In-Hospital Mortality

**Figure 1:**
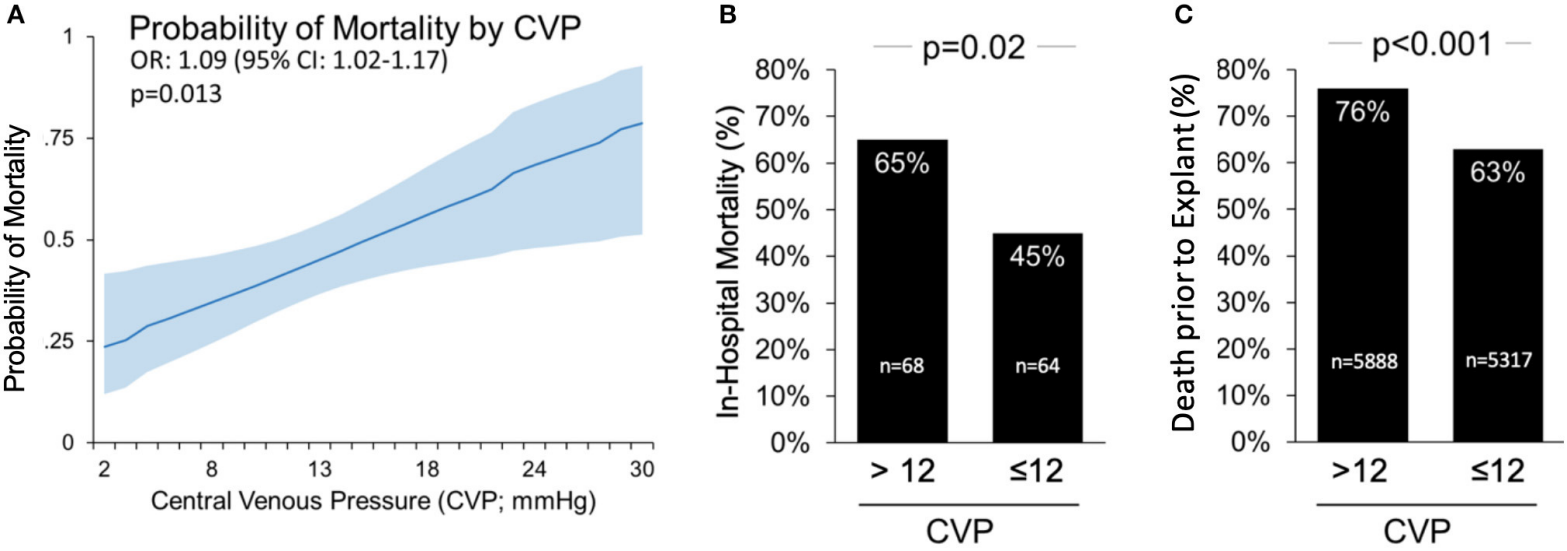
Central Venous Pressure (CVP) >12 mmHg on Impella support is associated with higher mortality in cardiogenic shock. **(A)** The probability of death based on CVP during left side Impella support; **(B)** CVP >12 is associated with higher in-hospital mortality rates among patients in the cVAD; and **(C)** associated with higher rate of death prior to device explant in the IQ Registry.

CVP was significantly higher among patients who died than among those who survived to discharge (14.0 ± 5.9 vs. 11.7 ± 4.6 mmHg, *p* = 0.014). The probability of in-hospital mortality increased directly with increased CVP measured during LV-MCS (Figure 1A). Receiver operating curve (ROC) analysis was performed to determine a cutoff point of CVP that best predicted mortality. The area under the receiver operating curve (AUROC) was 0.624 (95% CI 0.525–0.723). A CVP threshold of 12 was selected as the point of intersection between the sensitivity and specificity curves, with a Youden index of 0.196. Sensitivity of a CVP >12 to predict in-hospital mortality was 0.593 with a specificity of 0.602, positive predictive value 0.546, and negative predictive value 0.647. Using this cutoff, in-hospital mortality among patients with a CVP >12 was significantly higher than patients with CVP ≤12 (65 vs. 45%, *p* = 0.02, Figure 1B). To validate this analysis, we analyzed data from the IQ database and again found that death prior to device explant was significantly higher among patients with CVP >12 compared to those with CVP ≤12 (76 vs. 63%, *p* < 0.001, Figure 1C).

Univariate ORs and 95% confidence intervals are presented in Table 3. Among the variables tested, increasing age, decreasing LVEF, increasing CVP and need for mechanical ventilation were significantly associated with a higher odds of mortality. After adjusting for age, LVEF, and the need for mechanical ventilation, CVP remained significantly associated with in-hospital mortality (OR 1.10 per 1 mmHg increase in CVP, 95% CI 1.02–1.19, *p* = 0.013).

**Table 3:** Univariate and multivariate odds ratios.

**Variable**	**Odds ratio (95% CI)**	***P*-value**
**Univariate odds ratios**		
Age	1.03 (1.0–1.07)	0.047
Male sex	0.57 (0.24–1.37)	0.210
Hypertension	2.06 (0.9–4.8)	0.096
Diabetes mellitus	1.32 (0.6–2.7)	0.442
LVEF (%)	1.05 (1.0–1.1)	0.027
MAP (mmHg)	0.99 (0.97–1.0)	0.330
Cardiac Output (L/min)	0.81 (0.60–1.1)	0.151
CVP (mmHg)	1.09 (1.0–1.2)	0.015
Mechanical ventilation	2.55 (1.26–5.17)	0.009
**Multivariable odds ratios**		
Age	1.04 (1.0–1.1)	0.039
CVP (mmHg)	1.10 (1.02–1.19)	0.013
Mechanical ventilation	2.966 (1.40–6.29)	0.005

### CVP and Suction Events

**Figure 2:**
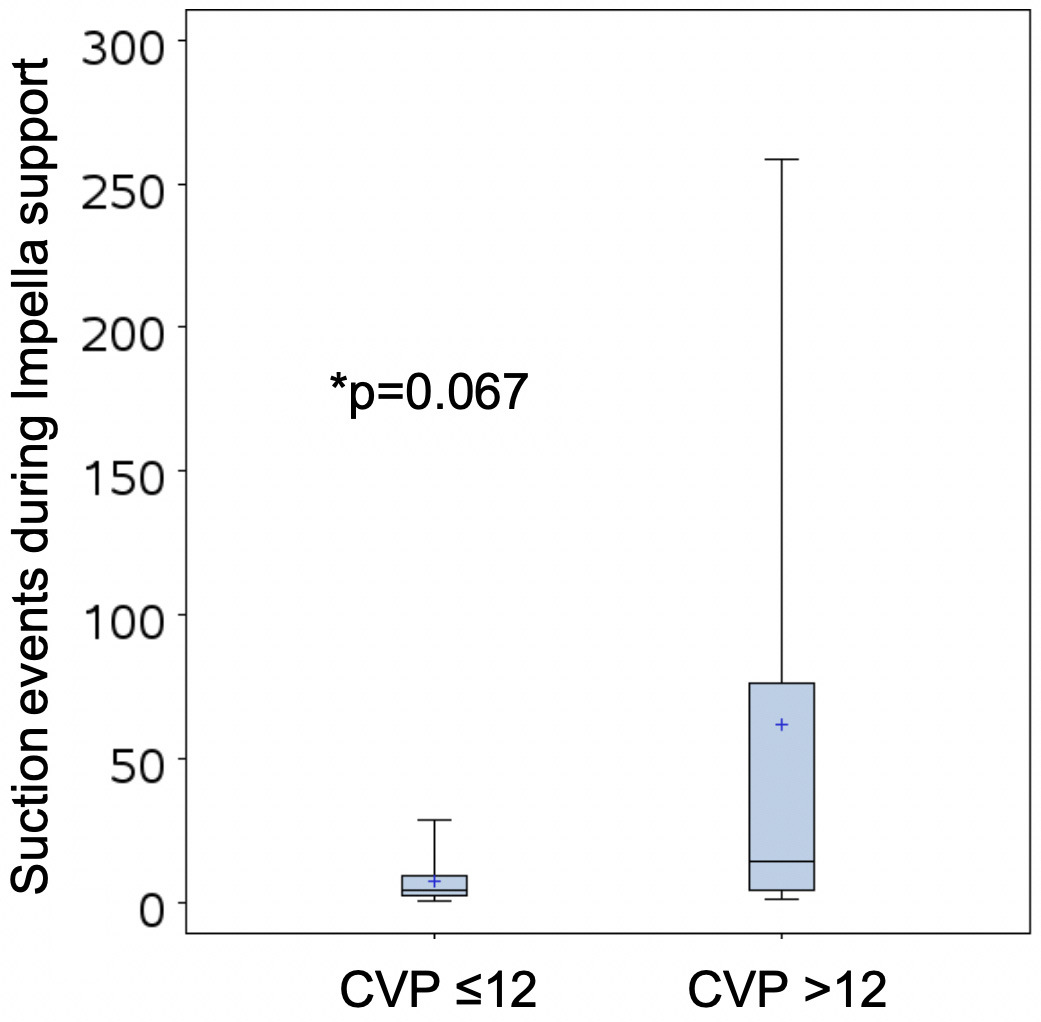
Suction events recorded by the Automated Impella Controller in patients with CVP >12 or ≤12.

We analyzed suction alarm data downloaded from the Automated Impella Controller (AIC) during Impella support, which were available in 21 out of 132 patients from the cVAD registry. Compared to patients with a CVP ≤12 during Impella support, suction events were more common among patients with a CVP >12 (62.11 ± 93.56 vs. 7.14 ± 8.79, number of events, *p* = 0.067, Figure 2).

## Discussion

We report for the first time that an elevated CVP during LV-MCS for cardiogenic shock is associated with in-hospital mortality. We identified that CVP was higher among patients who died in the hospital compared to those that survived to discharge in the cVAD registry. A CVP >12 among patients receiving LV-MCS predicted a higher odds of in-hospital mortality, even after adjustment for other variables. We further observed that suction events, which indicate reduced LV preload, were paradoxically more frequent among patients with a higher CVP, suggesting that a higher rate of impaired RV function may in part account for the higher short-term mortality observed among patients with high CVP. Collectively, these data suggest that identification of an elevated CVP during LV-MCS should trigger further evaluation of RV function with echocardiography or a pulmonary artery catheter.

While mechanical RV support devices such as the Impella RP can be used to stabilize patients with acute RVF, prompt recognition of RV dysfunction is paramount to prevent rapid deterioration and death. In contrast to LV failure where pulmonary edema is often readily apparent, right sided congestion indicating RV failure may be clinically silent, reinforcing the need for a high clinical suspicion and readily accessible bedside indicators which can be used to identify incipient RVF. Most well-validated hemodynamic indices of RVF such as the pulmonary artery pulsatility index (PAPi), CVP/PCWP ratio, and pulmonary vascular resistance (PVR) require use of a pulmonary artery catheter (PAC), and thus a more accessible bedside parameter is needed to trigger a formal evaluation for RVF.

On the basis of our findings, we propose that a CVP >12 in a patient receiving left sided mechanical support should prompt a formal hemodynamic and echocardiographic assessment of RV function to assess the need for decongestive therapies or RV mechanical support. The concurrent presence of frequent suction events in the face of adequate volume should further raise suspicion for RV pump failure. Prior studies including the Recover Right trial have proposed specific criteria for initiation of mechanical RV support including a CVP/PCWP ratio >0.63 or PAPi <0.9 in conjunction with echocardiographic indicators of RV dysfunction, though future studies will be needed to confirm the benefits of such an algorithm prospectively (3, 7).

Several limitations of our study must be acknowledged. First, these data are retrospective, and the limitations of cVAD data are such that the exact timing of laboratory and hemodynamic values relative to initiation of Impella support cannot be ascertained. Additionally, while we have proposed that the increased mortality observed in patients with high CVP is due at least in part to RVF, this connection cannot be definitively established due to a lack of high-resolution data on the specific causes of death among patients in this sample. Alternative causes of increased CVP that would also likely increase odds of mortality include hypervolemia, pulmonary hypertension, progressive LV failure, cardiac tamponade, renal failure, and the need for mechanical ventilation with high positive end-expiratory pressure. Accordingly, these results should be considered hypothesis generating, and warrant confirmation in larger, higher-resolution prospective studies. Finally, we did not have granular data on patient outcomes other than mortality, so some patients who survived in this analysis may have been bridged to durable VAD or transplant.

In conclusion, we report data from the cVAD registry showing that a CVP >12 predicts mortality in patients receiving left-sided aMCS and propose that a CVP >12 should prompt formal hemodynamic assessment for RV failure, especially in the presence of frequent suction events. Future studies will be needed to confirm these findings and refine hemodynamic criteria for mechanical RV support.

## Data Availability Statement

The raw data supporting the conclusions of this article will be made available by the authors, without undue reservation.

## Ethics Statement

The studies involving human participants were reviewed and approved by WIRB and Institutional IRBs for CVAD Registry. Written informed consent for participation was not required for this study in accordance with the national legislation and the institutional requirements.

## Author Contributions

EW generated figures and tables, and drafted the manuscript. KT assisted with generation of figures and editing of the manuscript. DB contributed to conception and design of the research as well as editing of the manuscript. NU and WO'N contributed to conception and design of the project. EO contributed to conception and design of the project, and editing of the manuscript. NK contributed to conception and design of the research, generation of figures and tables, and drafting/editing of the manuscript. All authors contributed to the article and approved the submitted version.

## Conflict of Interest

NK receives consulting/speaker honoraria and institutional grant support from: Abbott Laboratories, Abiomed Inc., Boston Scientific, Medtronic, LivaNova, MDStart, and Precardia. DB has received an unrestricted educational research grant from Abiomed Inc. Abiomed Inc. funded the study, provided data, and assisted with statistical analysis. They had no role in the interpretation of the data, decision to publish, or preparation of the manuscript. The remaining authors declare that the research was conducted in the absence of any commercial or financial relationships that could be construed as a potential conflict of interest.

## Abbreviations

CVP: central venous pressure
RHF: right heart failure
RVF: right ventricular failure
RR: Recover Right trial.
