# Supplementary material for: Central Venous Pressure and Clinical Outcomes During Left-Sided Mechanical Support for Acute Myocardial Infarction and Cardiogenic Shock
Source: Front Cardiovasc Med. 2020 Aug 28;7:155. doi: 10.3389/fcvm.2020.00155 (PMC7485579; doi:10.3389/fcvm.2020.00155)
Supplement: Supplementary file 1 [file Data_Sheet_1.docx]

**Table S1: Baseline Characteristics of study patients (Subjects with CVP during Impella support, n=132), compared to all patients meeting study inclusion criteria in the cVAD registry)**

| **Characteristics** | **All (N=387 Patients)** | **Subjects with CVP during Impella support (N=132 Patients)** | **Subjects without CVP during Impella support (N=255 Patients)** | **P-Value** |
| --- | --- | --- | --- | --- |
| Age |  |  |  |  |
| Mean±SD(N) | 63.85±11.55 (387) | 63.13±11.09 (132) | 64.23±11.79 (255) | 0.376 |
| BSA (m^2^) |  |  |  |  |
| Mean±SD(N) | 2.01±0.28 (375) | 2.02±0.23 (132) | 2.01±0.31 (243) | 0.843 |
| **Medical History** |  |  |  |  |
| Smoker | 58.86% (206/350) | 59.68% (74/124) | 58.41% (132/226) | 0.910 |
| Hyperlipoproteinaemia | 59.77% (211/353) | 57.94% (73/126) | 60.79% (138/227) | 0.651 |
| Hypertension | 76.92% (280/364) | 76.15% (99/130) | 77.35% (181/234) | 0.796 |
| Diabetes Mellitus | 45.03% (163/362) | 48.82% (62/127) | 42.98% (101/235) | 0.320 |
| CAD | 46.69% (169/362) | 50.00% (63/126) | 44.92% (106/236) | 0.378 |
| Stroke/TIA | 9.78% (35/358) | 6.40% (8/125) | 11.59% (27/233) | 0.137 |
| Renal Insufficiency | 16.76% (60/358) | 17.46% (22/126) | 16.38% (38/232) | 0.882 |
| Dialysis | 3.92% (14/357) | 1.59% (2/126) | 5.19% (12/231) | 0.151 |
| COPD/Chronic Pulmonary Disease | 14.77% (52/352) | 18.55% (23/124) | 12.72% (29/228) | 0.158 |
| Arrhythmia | 15.54% (55/354) | 18.55% (23/124) | 13.91% (32/230) | 0.282 |
| PVD | 9.30% (33/355) | 10.40% (13/125) | 8.70% (20/230) | 0.702 |
| CHF | 16.25% (58/357) | 19.05% (24/126) | 14.72% (34/231) | 0.297 |
| Valvular Disease | 7.32% (26/355) | 9.60% (12/125) | 6.09% (14/230) | 0.286 |
| Cardiomyopathy | 11.24% (40/356) | 12.80% (16/125) | 10.39% (24/231) | 0.487 |
| Prior MI | 27.03% (93/344) | 36.97% (44/119) | 21.78% (49/225) | 0.003 |
| LVEF (%) |  |  |  |  |
| Mean±SD(N) | 27.96±14.26 (185) | 25.10±12.79 (71) | 29.75±14.89 (114) | 0.031 |
| **Hemodynamics Prior to Impella Support** |  |  |  |  |
| HR (bpm) |  |  |  |  |
| Mean±SD(N) | 95.70±27.94 (337) | 96.05±26.84 (119) | 95.51±28.59 (218) | 0.865 |
| Mean Arterial Pressure (mmHg) |  |  |  |  |
| Mean±SD(N) | 81.38±21.85 (343) | 80.56±19.67 (117) | 81.80±22.93 (226) | 0.619 |
| Cardiac Index (L/min/m^2^) |  |  |  |  |
| Mean±SD(N) | 1.91±0.61 (48) | 1.92±0.54 (22) | 1.91±0.68 (26) | 0.946 |
| Cardiac Output (L/min) |  |  |  |  |
| Mean±SD(N) | 3.68±1.12 (48) | 3.73±1.12 (22) | 3.64±1.14 (26) | 0.799 |
| PCWP (mmHg) |  |  |  |  |
| Mean±SD(N) | 24.85±8.54 (40) | 26.50±11.20 (14) | 23.96±6.80 (26) | 0.449 |
| Pulmonary Artery Pressure Systolic (mmHg) |  |  |  |  |
| Mean±SD(N) | 37.08±11.08 (58) | 38.31±12.56 (27) | 36.00±9.68 (31) | 0.432 |
| LVEDP (mmHg) |  |  |  |  |
| Mean±SD(N) | 27.03±12.64 (34) | 28.36±10.25 (14) | 26.10±14.27 (20) | 0.616 |
| **Hematology and Blood Chemistry Pre-support** |  |  |  |  |
| RBC (10^6^/μL) |  |  |  |  |
| Mean±SD(N) | 4.38±2.60 (319) | 4.59±4.26 (110) | 4.27±0.87 (209) | 0.440 |
| WBC (10^3^/μL) |  |  |  |  |
| Mean±SD(N) | 14.37±8.06 (320) | 14.51±7.03 (110) | 14.30±8.57 (210) | 0.813 |
| Hgb (g/dL) |  |  |  |  |
| Mean±SD(N) | 13.02±6.00 (328) | 13.32±9.14 (113) | 12.86±3.35 (215) | 0.605 |
| Total Bilirubin (mg/dL) |  |  |  |  |
| Mean±SD(N) | 1.91±4.70 (185) | 1.61±4.64 (71) | 2.09±4.75 (114) | 0.495 |
| GFR (mL/min/m^2^) |  |  |  |  |
| Mean±SD(N) | 53.05±22.15 (265) | 54.81±21.68 (95) | 52.07±22.42 (170) | 0.336 |
| Lactate (mmol/L) |  |  |  |  |
| Mean±SD(N) | 6.19±4.99 (77) | 6.00±4.61 (36) | 6.35±5.35 (41) | 0.758 |

**Table S2: Admission and Procedural Characteristics of study patients (Subjects with CVP during Impella support, n=132), compared to all patients meeting study inclusion criteria in the cVAD registry)**

| **Characteristics** | **All**  **(N=387 Patients)** | **Subjects with CVP during Impella support (N=132 Patients)** | **Subjects without CVP during Impella support (N=255 Patients)** | **P-Value** |
| --- | --- | --- | --- | --- |
| Patient was transferred from another hospital | 33.33% (129/387) | 37.88% (50/132) | 30.98% (79/255) | 0.172 |
| Patient was supported with an IABP prior to Impella support | 15.58% (60/385) | 12.98% (17/131) | 16.93% (43/254) | 0.311 |
| Shock was present on admission | 48.84% (189/387) | 52.27% (69/132) | 47.06% (120/255) | 0.331 |
| Shock (Primary indication for Impella support) | 100.00% (387/387) | 100.00% (132/132) | 100.00% (255/255) | -- |
| Duration of shock |  |  |  |  |
| <6 hours | 65.27% (250/383) | 64.39% (85/132) | 65.74% (165/251) | 0.793 |
| 6-12 hours | 6.53% (25/383) | 4.55% (6/132) | 7.57% (19/251) | 0.255 |
| 12-24 hours | 5.48% (21/383) | 5.30% (7/132) | 5.58% (14/251) | 0.911 |
| >24 hours | 7.31% (28/383) | 9.85% (13/132) | 5.98% (15/251) | 0.166 |
| If Shock, patient experienced any of the following |  |  |  |  |
| Anoxic brain damage | 6.67% (21/315) | 4.72% (5/106) | 7.66% (16/209) | 0.323 |
| End-organ hypoperfusion | 21.17% (69/326) | 22.52% (25/111) | 20.47% (44/215) | 0.667 |
| Cardiac arrest | 52.20% (202/387) | 49.24% (65/132) | 53.73% (137/255) | 0.403 |
| If Shock, patient required any of the following |  |  |  |  |
| Mechanical Ventilation | 43.15% (167/387) | 50.00% (66/132) | 39.61% (101/255) | 0.050 |
| CPR | 46.51% (180/387) | 43.94% (58/132) | 47.84% (122/255) | 0.465 |
| STEMI | 73.51% (272/370) | 72.22% (91/126) | 74.18% (181/244) | 0.686 |
| NSTEMI | 26.49% (98/370) | 27.78% (35/126) | 25.82% (63/244) | 0.686 |
| Patient required inotropes or pressors prior to Impella support | 63.82% (247/387) | 69.70% (92/132) | 60.78% (155/255) | 0.084 |
| If yes, maximum number of different inotropes* |  |  |  |  |
| Mean±SD(N) | 2.11±1.13 (247) | 2.24±1.19 (92) | 2.03±1.09 (155) | 0.151 |
| Number of Vessels Treated (at least one lesion treated per vessel) |  |  |  |  |
| Mean±SD(N) | 1.64±0.76 (288) | 1.67±0.81 (103) | 1.63±0.73 (185) | 0.648 |
| Number of lesions treated |  |  |  |  |
| Mean±SD(N) | 2.02±1.25 (288) | 2.03±1.48 (103) | 2.02±1.11 (185) | 0.939 |
| Door to Impella Time (hours) |  |  |  |  |
| Mean±SD(N) | 31.67±61.97 (320) | 32.39±64.00 (113) | 31.28±60.99 (207) | 0.878 |
| Duration of Device Support (hours) |  |  |  |  |
| Mean±SD(N) | 78.42±78.45 (352) | 92.73±76.77 (123) | 70.73±78.43 (229) | 0.012 |
